# Synovial Cyst Mimicking an Intraspinal Sacral Mass

**DOI:** 10.1155/2014/953579

**Published:** 2014-03-04

**Authors:** Jason Hoover, Stephen Pirris

**Affiliations:** ^1^The Texas Brain and Spine Institute, 8441 State Highway 47, Suite 4300, Bryan, TX 77807, USA; ^2^Department of Neurosurgery, Mayo Clinic, 200 First Street SW, Rochester, MN 55905, USA; ^3^Department of Neurosurgery, Mayo Clinic, 4500 San Pablo Road, Jacksonville, FL 32224, USA

## Abstract

A 68-year-old female had a three-week history of severe low back pain radiating down the posterior left buttocks and left leg exacerbated by standing and walking. Lumbar spine MRI revealed cystic mass with similar intensity to cerebrospinal fluid located on dorsolateral left side of the sacral spinal canal inferior to the S1 pedicle. There was compression of left exiting S1 and traversing S2 nerve roots. Neurosurgery consult was requested to evaluate the cystic mass in the sacral spinal canal. After clinical evaluation, an unusually located synovial cyst was thought possible. Cyst contents were heterogeneous, suggestive of small hemorrhage and acute clinical history seemed reasonable. Left S1 and partial left S2 hemilaminectomy was performed and an epidural, partially hemorrhagic cyst was removed. There was no obvious connection to the ipsilateral L5-S1 facet joint. Pathology revealed synovial cyst, and the patient's leg pain was improved postoperatively. This synovial cyst was unusual as it had no connection with the facet joint intraoperatively and its location in the sacral canal was uncommon.

## 1. Case Presentation

### 1.1. Presentation and History

A 68-year-old female with a long history of intermittent back pain had a relatively acute onset of severe low back pain radiating down the posterior left buttocks and left leg for three weeks. She denied any trauma or other possible inciting factors. The pain had been getting progressively worse, and she also had developed numbness in the left S1 and S2 dermatomal distributions. An epidural steroid injection performed at a local facility provided partial relief and lasted for approximately 24 hours. The pain was described as intense, electrical pain different from any pain she had ever felt in terms of distribution and severity. Due to the exacerbating factors including standing and walking, thus, she presented to clinic in a wheelchair and preferred to lay in the fetal position. She had presented to the emergency department two days prior to neurosurgery consultation due to continued worsening of pain and numbness.

### 1.2. Physical Examination

A thorough neurological examination was negative except for a positive straight leg raise on the left.

### 1.3. Initial Diagnosis and Radiography

**Figure 1:**
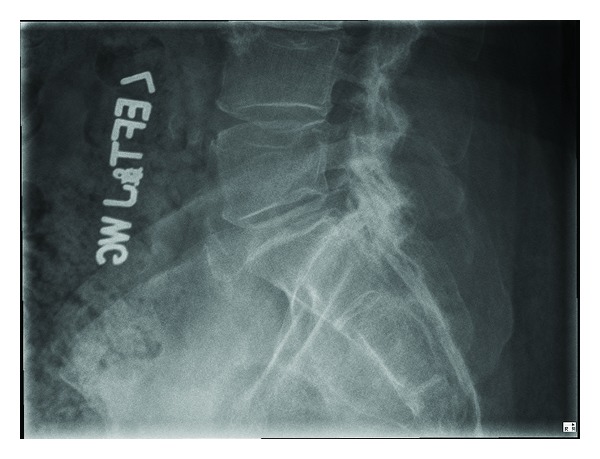
Lateral lumbar X-ray normal lumbosacral alignment and anatomy.

**Figure 2:**
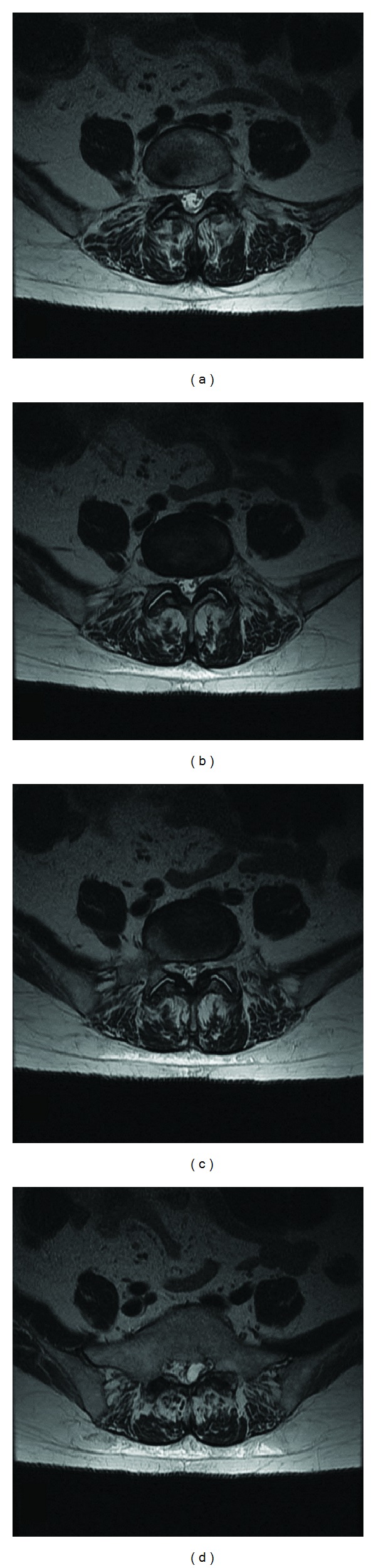
Axial T2-weighted MRI images showing sequential cuts ((a)–(d)) inferiorly from the L5-S1 facet joint showing an intrasacral mass separate from the L5-S1 joint.

**Figure 3:**
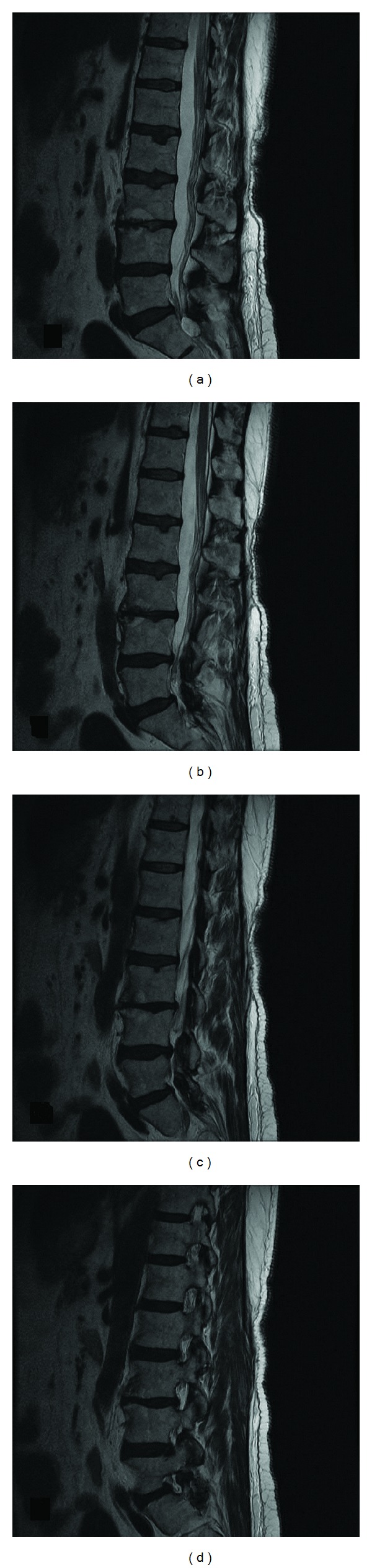
Sagittal T2-weighted MRI images showing sequential cuts ((a)–(d)) from midline to the foramen revealing the intrasacral mass separate from the left L5-S1 joint.

Lumbar spine X-ray did not reveal any abnormal alignment or transitional anatomy (Figure 1). Lumbar spine MRI (Figures 2 and 3) revealed a cystic mass with similar intensity to cerebrospinal fluid (T2 hyperintense/T1 hypointense) located on the dorsolateral left side of the sacral spinal canal inferior to the left S1 pedicle. It was causing significant compression on the exiting S1 and traversing S2 nerve roots. Sacral arachnoid or Tarlov cyst, cystic schwannoma, and synovial cyst were all included in the differential. She was referred to neurosurgery for urgent evaluation of her severe pain in the setting of an intraspinal sacral mass. After obtaining a clinical history from the patient and further reviewing of the MRI, the imaging diagnosis was questioned. A synovial (or juxtafacet) cyst was thought possible even though it was not in close proximity to the facet joint and was located in the sacral spinal canal. Further, the cyst contents were slightly heterogeneous and suggestive of small hemorrhage, and the acute clinical history was atypical for an arachnoid cyst or schwannoma. There was no connection to a facet joint noted on the imaging.

### 1.4. Clinical Recommendation and Outcome

**Figure 4:**
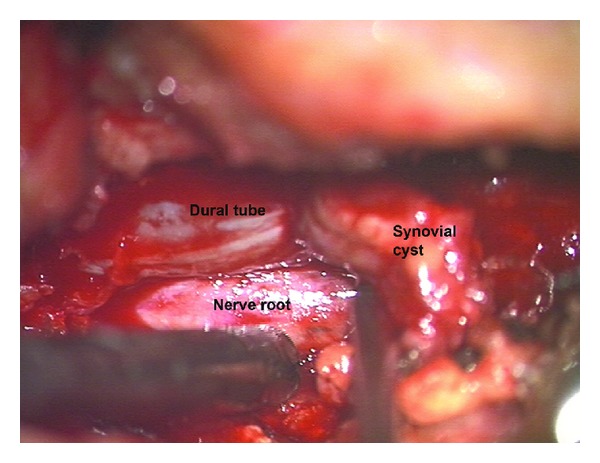
Intraoperative photograph of intraspinal sacral mass resection.

The patient was taken to surgery for a left S1 and partial left S2 hemilaminectomy. An epidural, partially hemorrhagic cyst inferior to the left S1 pedicle was dissected off the dura (Figure 4). The cyst was causing severe compression of the exiting and traversing nerve roots as seen on the preoperative MRI. There was no obvious connection to the left L5-S1 facet joint. Pathology revealed synovial cyst. The patient's left leg pain was significantly improved, and she was dismissed, on postoperative day two, neurologically stable.

## 2. Discussion

We present a case of a lumbar synovial cyst that on imaging and at surgery did not appear in proximity or continuous with the ipsilateral L5-S1 facet joint. This is rare, as these cysts are often found to be continuous with the facet joint of origin at surgery. The cyst resulted in adjacent nerve root compression and following cyst resection there was improvement in the radicular symptoms. The literature on unusually located lumbar synovial cysts is reviewed.

Lumbar synovial (or juxtafacet) cysts are a common cause of radicular pain due to their proximity to the exiting and traversing nerve roots. Low back pain is often related to degenerative changes in the lumbar facet joints. Many of these epidural cysts are found at the L4-5 level, presumably due to the greater degree of motion at this level, and to a lesser extent at the L5-S1 and L3-4 levels [1]. The cysts are typically found dorsolateral to the dural tube, resulting in posterior compression of the nerve roots, which contrasts with the typical location of a herniated disk which may be found ventral or ventrolateral to the dural tube and/or nerve root. The synovial cysts are found to be continuous with their facet joint of origin at surgery, but, in a large series of 19 patients, intra-articular dye injection demonstrated radiographic continuity in only three cases [2]. Synovial cysts are thought to arise due to hypermobility of the facet with herniation of synovium through an incompetent joint capsule [3]. On CT myelography, partial or complete interruption of intrathecal dye flow into the nerve root sleeve is observed. On MRI, synovial cysts can demonstrate hyperintensity on T2 series and hyperintensity between joint surfaces indicating intra-articular fluid in the facet. The synovial cysts may also be firm as evidenced by T2 hypointensity, and, if hemorrhage has occurred into the cyst, varying degrees of intensity can be seen on T1 and T2 series. Treatment for these lesions may include observation, with spontaneous resolution occurring in some, targeted facet joint, cyst, or nerve root injections for anti-inflammatory or pain-relief purposes, and/or surgical resection. In a large series of 194 patients with surgically treated lumbar synovial cysts, the average age was 66 years with the most common symptoms being painful radiculopathy and weakness, sensory loss, and decreased reflexes detected in 40%, 45%, and 57%, respectively. Overall, 22 patients underwent fusion either at the time of laminectomy and cyst resection or subsequently. Good pain relief and improvement in motor deficits were reported in 91% and 82%, respectively [4].

As stated above, while many lumbar synovial cysts are straightforward in presentation, appearance, and location, some are not. Métellus and colleagues [5] reported on a rare location of lumbar synovial cyst when they identified a three-centimeter cystic mass in the right L4 foramen that proved to be a synovial cyst impinging on the exiting L4 nerve root. They surmised the cyst arose from the right L4-5 facet joint even though it was not found to be in direct communication with the joint. They also found that the L4 nerve root was displaced inferiorly as opposed to superiorly as commonly seen with foraminal disk herniations [5]. Abdullah and colleagues [6] reported on four cases of synovial cysts that arose from the ligamentum flavum. Gheyi et al. [3] described the case of a midline lumbar synovial cyst. Palmieri et al. [7] described five unusual cases of “migrating” lumbar facet joint cysts in a seven-year period. They found that three of the cysts were located in the right S1 foramen, one in the right L5-S1 neural foramen, and one in the left paraspinal muscles between the levels of the L2–L4 spinous processes. The authors concluded that, while most synovial cysts abut the nearby facet joint, some can migrate from the joint of origin, and, therefore, a high level of suspicion must be maintained when formulating a diagnosis [7].

## 3. Conclusion

While lumbar synovial cysts often demonstrate continuity with the facet joint that may only be appreciated intraoperatively and not on preoperative imaging, they can also be remotely located. In our patient, the synovial cyst was found in the sacral spinal canal and distinctly separate from the facet joint. It is important to remember that epidural masses in the lumbosacral spine that present and image similar to synovial cysts may in fact be synovial cysts. Despite unusual locations of some of these synovial cysts, surgical resection remains a preferred treatment option.
